# Description of COVID-19 outbreaks in childcare facilities in Alberta, March 2020 to December 31, 2021

**DOI:** 10.14745/ccdr.v49i04a04

**Published:** 2023-04-01

**Authors:** Janis Geary, Sydney Rudko, Allison Scott

**Affiliations:** 1 Alberta Health, Edmonton, AB; 2 Public Health Agency of Canada, Edmonton, AB

**Keywords:** COVID-19, childcare, outbreaks, Alberta, surveillance

## Abstract

**Background:**

Children attending childcare are vulnerable to severe acute respiratory syndrome coronavirus 2 (SARS-CoV-2) infection, and mitigation measures such as masking, distancing, enhanced hygiene are not feasible for this population. Describing outbreak growth during the coronavirus disease 2019 (COVID-19) pandemic in childcare centres may provide insight into how to best mitigate the risks of COVID-19 and other infectious diseases in these settings. This article describes the childcare outbreaks and associated cases in Alberta at different time periods throughout the pandemic.

**Methods:**

Our observational analysis used data on outbreaks and associated cases tracked through the Alberta Health Services Communicable Disease Outbreak Management database. We included all COVID-19 outbreaks opened in childcare facilities (March 2020 to December 31, 2021). We compared the characteristics of outbreaks and cases during each wave of the pandemic.

**Results:**

There were a total of 841 childcare outbreaks, including 4,613 cases (70.2% in children and 29.8% adults). Many characteristics of outbreaks and cases varied across pandemic waves, including attack rates (12.1%–28.7% in adults and 5.8%–16.3% in children), percent of cases in children (56.4%–77.3%), and percent of outbreaks with a child index case (34.0%–70.1%). The overall average cases/outbreak was 5.5 (range: 1–68), and case examples of large outbreaks showed that delaying testing and attending daycare while symptomatic seemed to drive higher transmission.

**Conclusion:**

Waves had different outbreak and case characteristics, for both adults and children. Transmission may happen more readily among adults and among children than between those two groups. Measures shown to be effective to reduce transmission in other settings can be implemented here, such as vaccination, strictly enforcing the exclusion of those symptomatic, and facilitating rapid testing.

## Introduction

The Province of Alberta experienced five transmission waves of severe acute respiratory syndrome coronavirus 2 (SARS-CoV-2) infection, the causative agent of coronavirus disease 2019 (COVID-19), between March 2020 and March 2022. Early in the pandemic, children were reported to be less susceptible than adults to both infection and to severe outcomes such as hospitalization ((1,2)), with a high percentage having asymptomatic infection ((3)). Since then, much work has gone into understanding the infection in children ((4–8)), with studies noting that children are indeed at risk of infection, severe illness and long-term consequences ((9)), and are able to transmit the infection to others. Several studies have examined transmission and outbreaks in the childcare setting ((10–16)), some noting low transmission ((10,12,17,18)) or a higher attack rate or proportion of cases in adults ((13,14)). However, most of these studies examined transmission during the earlier phases of the pandemic in 2020 before more transmissible variants of concern (VOC) were circulating, and none compared outbreak characteristics in the same jurisdiction across different time points during the pandemic.

## Setting

The Province of Alberta has a population of 4.48 million ((19)). Statistics Canada estimated that 41% of Albertan children under the age of six years were enrolled in childcare between November 2020 and January 2021, and 54% between 2019 and 2020 ((20)). Childcare includes daycares, home-based care (“day homes”), preschools and out-of-school care. In these settings, children are in close contact with caregivers and other children in distinct cohort groups that typically have limited contact with other cohorts in the facility. Additionally, when public health restrictions do not otherwise limit cohort size, it is typical for facilities to group children and caregivers from multiple cohorts together, especially at drop-off and pick-up times, increasing the total number of individuals that interact. The staff-to-child ratios in childcare facilities range from 1:3 for infants, to 1:15 for children six years and older ((21)). Day homes are smaller groups and are limited to six children (not including the day home provider’s children), with not more than three children under three years of age ((21)).

Children attending childcare are vulnerable to acquiring SARS-CoV-2, largely because public health measures such as masking and distancing are not feasible for this group. Within this setting, the children eat meals in close proximity to multiple people every day and have poor hygienic practices ((22)). Additionally, for most of our analysis period, only a small proportion of their close contacts in the daycare setting was vaccine-eligible and young children continue to have low vaccination coverage. Even though all children six months and older have been eligible since August 2022, as of December 19, 2022, only 38.9% of children 5–11 years and 3.5% of children 6 months–4 years had two doses of a COVID-19 vaccine ((23)) and, due to current approvals for strain-specific boosters, it is likely children will continue to lag behind adults for some time.

From the beginning of the SARS-CoV-2 pandemic until December 31, 2021, childcare settings and those who attended them were often prioritized for public health measures, including increased tracking of outbreaks and contact tracing and availability of vaccines and polymerase chain reaction (PCR) testing. We detail these measures, along with others relevant to households, and the timelines they were implemented in the **Appendix**. These details may provide important context to understand the outbreaks, such as who was eligible for PCR testing at any given time. Documenting policy changes will assist future outbreak epidemiologists in understanding why we found the transmission patterns we did.

## Objective

Outbreaks of infectious illnesses in childcare facilities are not solely a phenomenon of the SARS-CoV-2 virus, as outbreaks of gastrointestinal and influenza-like illnesses have also been detected in these settings ((22)). The COVID-19 pandemic and the near-universal contact tracing brought about by the emergency provide interesting insights into the contextual factors that can exacerbate or mitigate the spread of infectious disease in childcare settings. Our objective is to provide aggregate surveillance data on outbreaks in childcare in Alberta in each pandemic wave through a descriptive statistical analysis of outbreaks and linked cases, and detailed case examples. Our summary description of all COVID-19 outbreaks tracked throughout the pandemic in Alberta is useful to public health practitioners interested in understanding the characteristics of such outbreaks in these settings and fills a gap in the literature on childcare outbreaks that will enable academic researchers to design more targeted analyses.

## Methods

Our surveillance period begins with the first identified case of COVID-19 linked to a childcare facility in Alberta in May 2020 and ends with the end of childcare outbreak tracking on December 31, 2021. During this time period, Alberta experienced five transmission waves, which we differentiated based on the dominant variants and the active case counts.

### Databases

We collected data on cases linked to outbreaks in childcare facilities using Alberta Health’s COVID-19 dataset that integrates data elements from several systems to provide a comprehensive COVID-19 cases and outbreak dataset. The Provincial Surveillance information system is a laboratory surveillance system that receives results for all notifiable diseases from laboratory surveillance conducted by Alberta Precision Labs ((24,25)). The communicable disease reporting system and the Communicable Disease Outbreak Management system contain information on COVID-19 cases and the data integration and measurement reporting system contains up-to-date information on people admitted and discharged from hospitals in Alberta (including for cases of multisystem inflammatory syndrome in children [MIS-C]). Reporting requirements changed throughout the pandemic and a detailed description of these requirements can be found in the Appendix. Vaccination data for cases was accessed from the Immunization and Adverse Reaction to Immunization database.

### Defining case and outbreak characteristics

We included only laboratory-confirmed COVID-19 cases. Throughout the pandemic, all cases were tested voluntarily, although the eligibility for PCR testing changed over time (see **Supplemental material**). The definition of an outbreak in a childcare setting also changed over time (see Supplemental material). All childcare outbreaks were handled by specialized outbreak contact tracing teams who worked with facility managers to identify cases, risks and plan mitigation strategies. Cases that attended a facility being tracked as an outbreak were linked via an outbreak investigation number. Throughout the pandemic, when an outbreak was identified in the childcare setting, exposed persons at the facility were notified, either required or recommended to quarantine, and encouraged to get PCR testing.

We identified probable outbreak index cases by their symptom onset date (SOD); cases with the same onset date were both counted as probable index cases (as such, there are more index cases than outbreaks). If a facility had two children with the same SOD it was categorized as “child index” and if it had two adults with the same SOD it was categorized as “adult index”. There were 39 outbreaks where both an adult and child had the same SOD, and these have been excluded when comparing outbreaks by index case age group. We defined each case’s infectious period as 48 hours prior to symptom onset, or 48 hours prior to a positive test result if the case was asymptomatic at the time of testing ((26)).

We report vaccination status of cases 12 years of age and older, as vaccines for children five years of age and older were not available until November 24, 2021. We considered a vaccine dose as valid if it was received 14 days or more before the SOD. We used vaccination data and the SOD to determine if the case was vaccinated at the time of their SOD, and we also reported if they were vaccinated by March 2022.

When reporting on severe outcomes, we included individuals who had a positive COVID-19 test at the time of hospitalization and children who were hospitalized at a later point for MIS-C. The MIS-C cases were reported by clinicians to the communicable disease team who provided weekly reports to public health surveillance partners.

Outbreaks were included in waves based on the date the outbreak was opened, and cases were included in waves based on the date the case was reported. Due to low numbers of cases linked to childcare outbreaks in wave 1, we combined waves 1 and 2 in our reporting.

### Statistics and descriptive analyses

We summarized the characteristics of cases linked to childcare outbreaks that were open over the course of the pandemic from March 2020 to December 31, 2021. We used R to analyze the data and conduct descriptive analysis, as well as Excel and Stata to conduct descriptive analysis and add summary variables. Contact tracers recorded information on the total populations of children and adults at risk in a facility, and attack rates were calculated as the number of lab-confirmed cases of COVID-19 out of the at-risk population. However, not all outbreaks that had the attack rate calculations performed by the investigators were precise, as there were 4/808 outbreaks (0.5%) reported as greater than 100%. These are likely errors. We did not identify a standard policy for how population at risk was defined.

### Outbreak case examples

We selected two outbreaks from each time period to examine outbreaks in these settings in more detail: from each time, we selected one of the largest outbreaks and one outbreak that had “average” characteristics (based on the number of cases, attack rate, duration and having an index case carrying the most common variant identified in that wave).

After extracting the cases linked to each of our identified outbreak, we manually searched the Communicable Disease Outbreak Management system for cases in the same household as each outbreak case. Household contacts were not included in attack rate calculations; they were included to demonstrate the context in which they arose and to describe the impact they had in the community. We used individual contact tracing notes and outbreak reports to describe the example outbreaks, although we obscured details to protect privacy. These data sources are often inconsistent and not all details about outbreak characteristics and dynamics are available for every case or outbreak. For example, few reports mention the proportion of staff in the facility who were vaccinated, and information about secondary transmission to cases not at the facility was inconsistently available throughout the pandemic.

Each outbreak is reported using the earliest symptom onset as “Day 1”, and each additional case is added based on when their symptoms started. Contact tracing in daycare settings ended early in wave 5 and we were not able to select a large and small outbreak for that wave; instead, we selected two outbreaks with confirmed Omicron cases.

## Results

**Figure 1:**
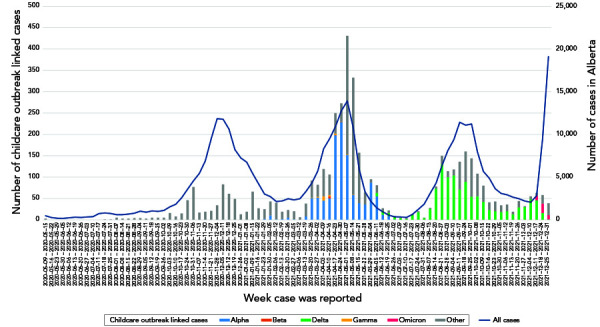
Epidemic curve of childcare associated COVID-19 cases compared to all cases in Alberta from May 2020 to December 2021^a^ Abbreviation: COVID-19, coronavirus disease 2019 ^a^ Cases are dated based on when the lab-confirmed case was reported to Alberta Health

The first childcare-associated case of COVID-19 in Alberta was identified in May 2020. Between then and December 31, 2021, there were 841 individual cases of COVID-19 at a childcare facility that met the definition of an outbreak and were tracked, with 4,613 associated cases. Of the 841 outbreaks, 63% (n=531) included at least one VOC case confirmed through screening or genotyping. The earliest outbreak due to a VOC SARS-CoV-2 variant was identified in January 2021 and was an Alpha variant (**Figure 1**). **Table 1** summarizes the characteristics of the outbreaks and **Table 2** details the average attack rate in each wave, for children and adults separately. Wave 3 stands out as having the highest number of outbreaks, the highest average cases/outbreak, the longest average duration and the highest average attack rates in both children and adults.

**Table 1:** Characteristics of childcare facility outbreaks opened during each wave of the COVID-19 pandemic, based on the date that the outbreak was opened

Characteristic	All	95% CI	Waves 1 & 2 May 10, 2020–Feb 25, 2021		Wave 3 Feb 26, 2021–Jul 1, 2021		Wave 4 Jul 2, 2021–Dec 15, 2021		Wave 5 Dec 16, 2022–Dec 31, 2022	
			n	95% CI	n	95% CI	n	95% CI	n	95% CI
Total outbreaks	841	N/A	144	N/A	336	N/A	331	N/A	30	N/A
Most common variant identified through genotyping	23.9% Alpha	22.7–25.1	95.0% Not genotyped	93.2–96.4	49.0% Alpha	46.9–51.1	67.3% Delta	64.9–69.7	33.8% Omicron	25.8–42.7
Average number of cases/outbreak	5.5	5.1–5.9	5.2	4.4–6.1	6.6	5.8–7.4	4.6	4.2–5.1	3.8	2.8–4.8
Percent of outbreaks with two or fewer cases	30.2	27.1–33.4	31.9	24.4–40.2	24.1	19.6–29.1	35.0	29.9–40.5	36.7	19.9–56.1
Outbreak average duration (days)^a^	9.7	9.0–10.4	9.1	7.2–11.1	10.3	9.2–11.5	9.4	8.4–10.4	9.0	5.7–12.3
Percent of outbreaks with a probable child index	55.1	51.6–58.5	34.0	26.3–42.4	48.5	43.1–54.0	70.1	64.8–75.0	63.3	43.9–80.1
Average percent of child cases	68.6	66.7–70.6	56.4	51.5–61.4	65.4	62.6–68.2	77.3	74.3–80.3	68.1	54.7–81.5
Average percent of child cases when probable index is an adult, N=339	46.8	44.1–49.6	43.2	37.8–48.6	49.7	45.9–53.4	47.4	41.1–53.6	31.7	12.1–51.4
Average percent of child cases when probable index is a child, N=463	85.4	83.7–87.1	80.8	73.8–87.9	80.4	77.4–83.4	89.6	87.5–91.7	89.1	80.5–97.7
Percent of index cases tested within one day of symptom onset, N=968	40.0	36.9–43.1	51.4	43.7–59.1	40.6	35.7–45.6	34.6	29.8–39.7	30.8	15.9–52.4

Abbreviations: CI, confidence interval; COVID-19, coronavirus disease 2019; N/A, not applicable

^a^ Calculated as the number of days between symptom onset of first case and last case

**Table 2:** Attack rates in children and adults stratified by the age category of the index case

Characteristic	All	95% CI	Waves 1 & 2 May 10, 2020–Feb 25, 2021		Wave 3 Feb 26, 2021–Jul 1, 2021		Wave 4 Jul 2, 2021–Dec 15, 2021		Wave 5 Dec 16, 2022–Dec 31, 2022	
			n	95% CI	n	95% CI	n	95% CI	n	95% CI
Average % attack^a^	13.4	12.2–14.6	12.6	10.2–14.9	18.2	15.8–20.6	9.6	8.3–10.9	7.5	4.9–10.1
Average % attack in only children	11.8	10.6–13.0	8.9	7.1–10.7	16.3	14.0–18.7	9.1	7.7–10.6	5.8	3.4–8.2
Average % attack in only children when probable index is an adult, N=314	10.4	8.8–12.0	8.5	6.1–10.8	13.3	10.3–16.3	7.9	5.6–10.3	4.9	-0.1–9.9
Average % attack in only children when probable index is a child, N=457	12.3	10.7–13.9	9.7	6.6–12.9	18.3	14.6–21.9	9.2	7.5–10.9	6.3	3.5–9.1
Average % attack in only adults	21.4	19.3–23.6	28.5	21.4–35.5	28.7	25.0–32.4	12.1	9.9–14.3	14.3	6.9–21.6
Average % attack in only adults when probable index is an adult, N=314	32.6	28.9–36.4	37.3	26.7–47.9	35.0	30.5–39.5	25.2	19.1–31.3	25.7	11.5–39.9
Average % attack in only adults when probable index is a child, N=457	12.8	10.4–15.2	13.2	6.6–19.7	22.6	16.8–28.4	6.4	4.7–8.0	7.6	0.0–15.2

Abbreviation: CI, confidence interval

^a^ 33 outbreaks did not have information on the total population at risk, so attack rates could not be calculated

**Table 3** summarizes the characteristics of cases associated with the outbreaks and Figure 1 is an epidemiologic curve of all childcare outbreak linked cases compared to the province-wide cases. Overall, cases in the childcare setting peaked along with cases in the community. Wave 4 had the highest proportion of cases being children. There were no deaths in our population, 0.4% of adults were admitted to the intensive care unit and 2.2% of adults and 0.3% of children were admitted to hospital. As there were so few cases of MIS-C, we did not report these separately from other types of childhood hospitalization.

**Table 3:** Characteristics of cases linked to any childcare facility outbreak during each wave of the COVID-19 pandemic, based on the date the case was reported^a^

Characteristic of cases	All	95% CI	Waves 1 & 2 May 10, 2020–Feb 25, 2021		Wave 3 Feb 26, 2021–Jul 1, 2021		Wave 4 Jul 2, 2021–Dec 15, 2021		Wave 5 Dec 16, 2022–Dec 31, 2022	
			n	95% CI	n	95% CI	n	95% CI	n	95% CI
All (N)	4,613	N/A	758	N/A	2,207	N/A	1,518	N/A	130	N/A
**Age (%)**										
Younger than 1 year	0.3	0.2–0.5	-	N/A	-	N/A	0.2	0.0–0.6	0	N/A
1–2 years	18.5	17.4–19.7	17.2	14.5–20.0	21.9	20.2–23.7	15.0	13.2–16.8	10.8	6.0–17.4
3–4 years	27.8	26.5–29.1	24.9	21.9–28.2	26.4	24.5–28.3	31.7	29.4–34.1	22.3	15.5–30.4
5–9 years	21.0	19.9–22.3	13.6	11.2–16.2	17.4	15.8–19.0	29.1	26.8–31.5	32.3	24.4–41.1
10–19 years	2.6	2.1–3.1	3.6	2.4–5.1	1.7	1.2–2.3	3.4	2.6–4.5	-	N/A
20–29 years	7.6	6.8–8.4	10.3	8.2–12.7	7.7	6.7–8.9	5.8	4.7–7.1	10.0	5.4–16.5
30–39 years	8.6	7.8–9.4	12.4	10.1–15.0	8.6	7.5–9.9	6.6	5.4–8.0	8.5	4.3–14.6
40–49 years	8.3	7.6–9.2	12.3	10.0–14.8	9.4	8.2–10.7	4.7	3.7–5.9	9.2	4.9–15.6
50–59 years	4.3	3.7–4.9	4.2	2.9–5.9	5.3	4.4–6.4	2.8	2.0–3.7	4.6	1.7–9.8
60 years and older	1.0	0.7–1.4	1.5	0.7–2.6	1.1	0.7–1.6	0.7	0.3–1.3	-	N/A
Child (younger than 18 years)	69.1	67.7–70.4	57.4	53.8–60.9	67.1	65.1–69.0	78.1	75.9–80.1	66.9	58.1–74.9
Adult (18 years and older)	30.9	29.6–32.3	42.6	39.1–46.2	32.9	31.0–35.0	21.9	19.9–24.1	33.1	25.1–41.9
**Sex^b^ (%)**										
Children: female	47.3	45.5–49.0	48.7	43.9–53.5	46.8	44.2–49.3	47.8	45.0–50.7	41.4	30.9–52.4
Children: male	52.7	50.9–54.4	51.3	46.5–56.1	53.2	50.6–55.7	52.2	49.3–55.0	58.6	47.6–69.1
Adults: female	96.0	94.9–97.0	96.3	93.6–98.1	95.9	94.2–97.2	97.0	94.5–98.6	88.4	75.0–96.1
Adults: male	3.9	3.0–5.1	3.7	1.9–6.4	4.0	2.7–5.7	3.0	1.4–5.5	11.6	3.9–25.1
**Genotype (%)**										
Alpha	23.9	22.7–25.1	2.6	1.6–4.0	49.0	46.9–51.1	0	N/A	0	N/A
Beta	0.2	0.1–0.3	0	N/A	0.3	0.1–0.7	0	N/A	0	N/A
Gamma	0.5	0.3–0.7	0	N/A	1.0	0.6–1.5	0	N/A	0	N/A
Delta	23.4	22.2–24.6	0	N/A	1.3	0.9–1.9	67.3	64.9–69.7	20.8	14.2–28.8
Omicron	1.1	0.8–1.4	0	N/A	0	N/A	0.3	0.1–0.8	33.8	25.8–42.7
Not genotyped	39.0	37.5–40.4	95.0	93.2–96.4	29.4	27.5–31.3	25.0	22.9–27.3	37.7	29.3–46.6
Unresolved or wild type	12.1	11.2–13.1	2.4	1.4–3.7	19.0	17.4–20.7	7.3	6.1–8.7	7.7	3.8–13.7
**Vaccine doses at SOD (%)^c^**	**N=1,433**		**N=325**		**N=731**		**N=344**		**N=43**	
1 dose	5.1	4.0–6.4	0	N/A	5.6	4.1–7.6	9.6	6.6–13.3	0	N/A
2 doses	12.1	10.4–13.9	0	N/A	-	N/A	40.4	35.1–45.9	86.0	72.1–94.7
3 doses	-	N/A	0	N/A	0	N/A	-	N/A	-	N/A
No doses	82.6	80.6–84.6	100.0	98.9–100^d^	94.3	92.3–95.8	49.7	44.2–55.2	-	N/A
**Vaccine doses as of March 2022 (%)^c^**	**N=1,433**		**N=325**		**N=731**		**N=344**		**N=43**	
1 dose	2.0	1.4–2.9	1.5	0.5–3.6	1.1	0.5–2.1	4.8	2.8–7.7	0	N/A
2 doses	57.4	54.8–60.0	54.5	48.9–60.0	58.1	54.4–61.7	59.6	54.1–64.9	51.2	35.5–66.7
3 doses	31.5	29.1–34.0	35.7	30.5–41.2	35.4	32.0–39.0	17.4	13.5–21.9	44.2	29.1–60.1
4 doses	-	N/A	0	N/A	0	N/A	-	N/A	-	N/A
No doses	8.9	7.4–10.5	8.3	5.5–11.9	5.3	3.8–7.2	18.0	14.0–22.5	-	N/A
**Severe outcomes in children (%)**	**N=3,187**		**N=435**		**N=1,480**		**N=1,185**		**N=87**	
Hospitalized	0.3	0.1–0.5	-	N/A	-	N/A	-	N/A	-	N/A
Intensive care unit	0	N/A	0	N/A	0	N/A	0	N/A	0	N/A
Death	0	N/A	0	N/A	0	N/A	0	N/A	0	N/A
**Severe outcomes in adults (%)**	**N=1,426**		**N=323**		**N=727**		**N=333**		**N=43**	
Hospitalized	2.2	1.5–3.2	1.5	0.5–3.6	2.2	1.3–3.5	3.3	1.7–5.8	0	N/A
Intensive care unit	0.4	0.1–0.8	-	N/A	-	N/A	-	N/A	-	N/A
Death	0	N/A	0	N/A	0	N/A	0	N/A	0	N/A

Abbreviations: CI, confidence interval; COVID-19, coronavirus disease 2019; N/A, not applicable; SOD, symptom onset date; -, proportion was being suppressed because the count was <5

^a^ Proportions based on case counts that are fewer than five are suppressed (indicated with a dash)

^b^ There were fewer than 1% of individuals who did not disclose or for whom biological sex was missing in the database

^c^ This section only includes the 1,433 cases that were 12 years of age and older

^d^ This is a one-sided test, 97.5% confidence interval

### Outbreak case examples

Our case examples are detailed in **Table 4**. We did not include some potentially identifying information, such as the geographic zone the facility is in, precise dates of the outbreak, precise facility size or counts of severe outcomes. The “Day 1” for each outbreak is the earliest symptom onset set. These eight outbreaks included 143 cases that attended childcare facilities and an additional 44 who lived in the same household and had symptoms after those of the childcare case. These 187 total cases included fewer than five hospitalizations and intensive care unit admissions and no deaths.

**Table 4:** Details of example childcare facility outbreaks from each wave of the pandemic

Wave	Size of facility and number of cohorts with cases	Genotypes	Public health measures described	Information about index case	Number of cases at the facility	Subsequent cases in households of childcare attendees	# of cases who attended facility while infectious/on SOD/after SOD	Other information^a^
Wave 1 & 2A	Unknown Three cohorts	Likely wild type (no genotyping)	Staff reported the centre follows sanitization, distancing, staff masking, and a limit of two staff in break rooms at a time.	A staff member who worked while infectious (but symptoms developed after work) in the cohort that later had child cases.	22 from 21 households; 16 children, 6 adults	Three households with child index (4 cases). One household with adult index (1 case).	19/8/0	By the time the outbreak was reported on Day 8, almost all cases had already developed symptoms. Cases in children seem limited to a single cohort, while staff cases span at least three. The facility was closed on Day 6 due to cases.
Wave 1 & 2B	Fewer than 50 Two cohorts	Likely wild type (no genotyping)	Staff at the facility reported continuously masking, cohorting children, increased cleaning, and limiting the number of children at activities.	Two staff who both could be index cases were tested while asymptomatic, but worked while infectious and pre-symptomatic.	Five from 4 households; 2 children, 3 adults	One household with child index (1 case).	4/1/0	Cases in children were limited to a single cohort. The facility closed on Day 9, but exposed staff and children were quarantined prior to this.
Wave 3A	More than 100 ~90% of cohorts	66/74 genotyped were Alpha variant 1/74 is presumptive wild type 7/74 were unresolved	The facility conducted symptom and temperature screening, enhanced cleaning and staff wore face masks when not in their cohort. Staff had a shared bathroom and lunchroom; however, the lunchroom was reported to be in use by only one person at a time.	A child who attended on but not after SOD. Second case was a staff member working in multiple cohorts while symptomatic.	69 from 61 households; 46 children, 23 adults	Six households with child index (7 cases). One household with child and adult index (1 case). Nine households with adult index (15 cases).	29/12/4	Many of the earlier cases did not get tested quickly after symptom onset, including a staff who worked after their SOD and in multiple cohorts. The outbreak was not flagged for tracking until 10 days after the first case developed symptoms, at which point to 17 individuals at the facility had already developed symptoms.
Wave 3B	Fewer than 50 One cohort	12/13 genotyped were Alpha variant 1/13 was unresolved	Staff reported measures in place including hand hygiene, masking, frequency surface cleaning and physical distancing. Staff are limited to only two at a time in the lunchroom.	The likely index is a staff member, who did not get tested for six days after symptom onset.	Six from 6 households; 3 children, 3 adults	Two households with child index (3 cases). Two households with adult index (6 cases).	3/2/0	The facility closed on Day 7 of the outbreak. Vaccines were not yet widely available and none of the staff cases had received any doses yet, nor had vaccine-eligible household cases.
Wave 4A	More than 100 Two cohorts of children, unknown staff cohorts	19/20 genotyped were Delta variant 1/20 was unresolved	Facility reports public health measures including distancing the children, when possible, enhanced surface cleaning, daily health checks and continual staff masking.	The earliest case was a child with no known household exposures who did not get tested until four days after symptom onset.	20 from 18 households; 17 children, 3 adults	Three households with child index (3 cases).	20/9/3	The outbreak seems to have spread from one cohort to the other via siblings. The staff cases all had at least one vaccine dose, with 2/3 having had two doses. 5/9 vaccine-eligible household cases had two doses. The remaining had no doses. The facility closed on the evening of Day 7.
Wave 4B	50–100 Two cohorts	5/5 genotyped were Delta variant	Staff reported using enhanced surface cleaning and hand hygiene, and daily symptom checks. Staff had not been masking prior to the outbreak but began masking again.	Likely index was a child who attended after their symptoms began and who did not get tested until four days later.	Five from 5 households; 3 children, 2 adults	No observation of transmission from a daycare case to a household member.	5/1/2	One staff case had no vaccine doses, and the other had two. No indication that the facility closed at any point during the outbreak.
Wave 5A	50–100 Two cohorts	7/8 genotyped were Omicron variant 1/8 was unresolved	Both staff and children were reported to wear masks, and staff conducted enhanced cleaning throughout the facility. Children and staff ate together in their cohorts.	Child who did attend just before but not on or after SOD, but did not get tested until four days after SOD.	Eight from 6 households; 7 children, 1 adult	One household with a child index (1 case).	8/3/4	All vaccine-eligible child cases had received one dose of the vaccine, although two had received that dose just a few days prior to their SOD. The one staff case had two doses. Vaccine-eligible household cases had 1–2 doses. The facility closed on Day 5.
Wave 5B	50–100 One cohort	5/7 genotyped were Omicron variant 2/7 were Delta variant	No information about public health measures at the facility were documented in available records.	The Delta index case was a staff who attended after their SOD. The Omicron index case was a staff who attended after their SOD. The staff member’s household was exposed at an event, but the staff did not believe their core symptom^b^ related to COVID-19 and did not get tested for four days.	Eight from 7 households Delta: 1 child, 1 staff Omicron: 4 children, 2 staff	One household with a child index (2 cases).	8/4/4	Staff cases all had two vaccine doses. Three children had one dose, and two had no doses. Vaccine-eligible household members had two doses. The facility closed on Day 26.

Abbreviations: COVID-19, coronavirus disease 2019; SOD, symptom onset date

^a^ Here we included additional information gathered somewhat unsystematically from the outbreak investigation or comments from staff and parents

^b^ Core symptom refers to fever, cough, sore throat or shortness of breath

## Discussion

Our surveillance of COVID-19 outbreaks in Alberta throughout the first two years of the pandemic is an important contribution to knowledge of transmission in this setting. Previously, reporting in Alberta was limited to lists of childcare outbreaks that reached a specific threshold, or cases in age groups that would also include children in care. Additionally, the existing literature on outbreaks in childcare focuses on specific facilities or time periods. Here, we discuss differences across time periods and age groups, the evidence of the impacts of vaccination in this setting, and the factors that appeared to drive the large outbreaks we examined in detail.

We found that the outbreak and associated case characteristics we examined often differed for each time period. The number of index cases tested within one day of symptom onset dropped steadily through the time periods (51.4%, 40.6%, 34.6% and 30.8%, from May 2020 to December 31, 2021), which could be due to cases either choosing not to seek testing or cases not being able to access timely testing. Wave 3 had the lowest proportion of outbreaks with limited secondary transmission (i.e. transmission beyond the first case(s) who were identified and initiated outbreak tracking). The attack rates in adults dropped from 28.5% in waves 1 and 2 and 28.7% in wave 3 (when vaccines were largely unavailable), to 12.1% and 14.3% in waves 4 and 5, respectively. The attack rates in children varied somewhat less across time periods and was highest in wave 3 (16.3%) and 8.9%, 9.1% and 5.8% in waves 1, 2, 4 and 5, respectively. Notably, in wave 4, when vaccination coverage in the adult population was high and immune-evasive variants like Omicron had not yet begun to dominate in Alberta, the percent of outbreaks with children-index cases was highest (70% vs. the pandemic average of 55.1%) and the attack rate in adults was also the lowest (12.1% vs. the pandemic average of 21.4%). The highest proportion of cases in children (77.3%) was also in wave 4. The only scenario when the attack rate was higher in children than adults was when the probable index was also a child in wave 4.

Our data analysis suggests that, at least in the childcare setting, spread from children to adults and adults to children is less common than spread within each of these groups. The proportion of cases in children is at its highest when children are the probable index (85.4%), compared to only 46.8% when an adult is the index case. This contrasts with a 2020 article, where Ehrhardt *et al.* described child-to-child transmission in schools/childcare facilities as “very uncommon” ((10)). Link-Gelles *et al.* noted limited secondary transmission in childcare, finding that 69% of programs had a single case with no apparent secondary transmission ((17)). While we were only able to look at the proportion of outbreaks with limited spread (only 1–2 total cases), as facilities with only a single case were not tracked after August 2020, it is still striking that we found only 30.2% of outbreaks with limited spread. Adults are generally overrepresented in the case counts as well. Our work found that the average outbreak cases were 68.6% children and 31.4% adults, which is likely an over-representation of adult cases as, due to required ratios of staff to children in childcare, adults providing care are generally 25% or less a childcare cohort (one staff per three infants) and as low 6% in older groups (one staff per 15 children). Kim *et al.* also reported a high proportion of cases in adults ((13)). Adults often had a much higher attack rate than children, averaging 21.4% across the pandemic vs. 11.8% in children. The highest average attack rate for adults was in waves 1 and 2 when 37.3% of adults were infected when there was an adult index case at the facility. In the same scenario, the attack rate in children was only 8.5%. The highest attack rate children experienced was in wave 3 (18.3%, when a child was the index case). In 2022, Li *et al.* reported that staff had a much higher attack rate than children (47% vs. 11%), in line with our observations ((14)), although in 2021 Loenenbach *et al.* reported more similar attack rates in children vs. adults (23% vs. 30%) ((15)). Lopez *et al.* identified similarly low attack rates for facilities in 2020, between 17% and 18% for the entire facility ((27)).

Our observations also point to the value of vaccination in these settings. While wave 4 was dominated by the more contagious and more virulent Delta variant, 40% of vaccine-eligible cases had two doses of the vaccine, which may have helped limit spread. Wave 4 had a lower number of average cases/outbreak than wave 3 (when vaccines were still largely unavailable) and the lowest attack rate in adults. There is a stark contrast between outbreaks in waves 1 and 2 when an adult was the index case and no one was vaccinated (attack rate is 37.3%) and wave 4 when a child was the index case and much of the adult population was vaccinated (6.4%), suggesting that the most transmission in the childcare setting happens when unvaccinated adults introduce the virus into an unvaccinated population, and the least transmission happens when a child introduces the virus into a population where adults are at least mostly vaccinated. Wave 4 had a much higher proportion of outbreaks with a probable child index case (70.1% in wave 4 compared to 48.5% in wave 3), indicating that when vaccination coverage was high, adults were transmitting in these settings less frequently. At the beginning of wave 4 in early July 2021, the coverage of one dose of the vaccine in the Alberta population aged 12–19 years was 62.7%, 20–39 years was 63.9% and 40–59 years was 75.6%, and this rose to 85.4%, 83.9% and 88.9%, respectively, by December 15. The percent of childcare cases in wave 4 who had no doses of vaccine appears higher than the general Alberta population. While we found that 49.7% of cases 12 years of age and older in wave 4 had no doses of vaccines at their SOD, the percent of those aged 12–19, 20–39 and 40–59 years in the general population with no vaccine doses was 37.3%, 36.1% and 24.4%, respectively. This dropped by the end of wave 4 to 14.6%, 16.1% and 11.1%, respectively. While we cannot directly compare the Alberta general population vaccination rates to our observation that 49.7% of cases were unvaccinated, which is for the entire time period, combining all of those 12 years and older and are specific to SOD. There still appears to be an over-representation of individuals with no vaccine doses in our cases compared to what would have been expected at the time, which indicates that unvaccinated adults were more susceptible to infection.

As expected from previously published articles, most COVID-19 cases in our adult population were female, which aligns with typical staffing at childcare centres. Again, as with previously published articles, our work also noted that cases in childcare facilities trend with cases in the community ((13,17)) and the most common variants identified via genotyping aligned with what was circulating in the community at the time.

There are a few things to note from the more detailed contextual factors in the case examples described in Table 4. The largest outbreaks involved either individuals attending while symptomatic (waves 3, 4 and 5) or, in the case of waves 1 and 2, individuals delaying testing for several days after symptom onset, which prevented contact tracing and isolation, and prevented parents from being able to make informed decisions about risks of sending their child. There are no data on when specific symptoms began, so it is not possible to identify if children were attending their childcare facilities with symptoms that were mandated to require testing. We do not have information available about air quality at the facilities and cannot determine what role ventilation may have had in increasing or decreasing spread in each outbreak. While contextual information, such as our case examples provide, can be difficult to interpret and enable few conclusions to be drawn, the experiences of what occurs on the ground in an outbreak situation can be important lessons for policy-makers and operators alike.

While our data cannot be used to estimate how often a child or adult infected in childcare settings infects their household members, they do demonstrate that household transmission occurs from both adults and children. Of the 127 households that had cases linked to any of the case examples, 30 had confirmed cases with symptoms that started after the case linked to a childcare facility (17 child household index cases, 12 adult index cases and one household with both an adult and a child index case). This contrasts with studies that suggest no secondary transmission from school-aged children ((28)) and reduced transmission from children to their households ((29)). Our findings of increased cases in children when a child is the index case contrast with the Silverberg *et al.* article ((29)) which reported that children transmit to other children less readily but is consistent with an article by Paul *et al.* ((30)) that suggested that children aged 0–3 years were the most at risk for transmitting to their households.

### Strength and limitations

A major strength of our analysis is the inclusion of all cases linked to outbreaks at childcare facilities in the Province of Alberta. There are, however, still several limitations to our work. The eligibility for PCR testing, the definition of outbreak and the nature of contact tracing changed over time. Our analysis includes cases who chose to seek testing and this sample may have missed cases who did not wish to get tested. In examining individual outbreaks, there are several major limitations that require great caution in any attempts to correlate outcomes of the outbreak with any specific behaviours or public health interventions. The specific public health measures implemented in each facility were not systematically captured and there was no way to verify that the measures are being implemented consistently. Some descriptions of measures were provided by individual staff members and not the official outbreak investigation, which sometimes did not indicate what measures were in place. Index cases were identified through self-reported symptom onset data and individuals might not have accurately remembered when mild symptoms started, might have disregarded symptoms that do not seem to fit and (with very young children) might have had earlier symptom onset than when they were able to communicate them to their care providers. We identified household cases of childcare cases through contact tracing data, which may be incomplete. We only have information on those who tested positive via PCR testing—there may have been additional cases who tested positive via rapid test and therefore did not seek PCR testing. We do not know if exposed individuals got PCR testing and were negative, or if they did not get any PCR testing and may have been infected. We also do not know the vaccination status of exposed individuals, except for comments on vaccination coverage of staff in outbreak reports.

The total cases linked to wave 5 outbreaks are likely underestimated because testing in wave 5 became limited just a week into the wave due to testing capacity issues. Many childcare facilities closed shortly after wave 5 began, due to the Christmas holiday, and childcare outbreak tracking ended shortly after. Our estimates for wave 5 are also imprecise due to the resulting low number of outbreaks and associated cases. It is possible that patterns observed with earlier outbreaks would have changed with the introduction of the Omicron variant.

## Conclusion

While attack rates were lower in children than in adults in childcare facilities, children were at risk of COVID-19 infection in this setting and could transmit to caregivers and each other. The average attack rates in adults and the high proportion of cases in adults indicated that spread happened more readily among adults than children. Measures known to prevent spread among adults may be an important factor in preventing spread across childcare cohorts, and in keeping facilities open during outbreaks. Almost half of all childcare cases have been in children under five years of age; a population that has only recently been eligible for vaccination, will likely be delayed due to ineligibility for future variant-specific boosters, and is unable to mask properly or physically distance from caregivers and peers. As community transmission continues in Alberta and around the globe, so does the potential for emergence of new variants. It is unclear what role the public health measures, such as masking among staff, played in preventing spread to children and how important such measures will be in future pandemic waves. However, measures shown to be effective in other settings will likely be effective here, and childcare facilities can implement protections such as encouraging vaccination among their staff, eligible children and their families, strictly enforcing the exclusion of symptomatic staff and children, and facilitating rapid testing of those who become symptomatic. As each wave in our analysis had unique characteristics, facility managers should be aware that as new variants/subvariants emerge, there may be changes in transmission dynamics and therefore changes in which measures are most effective.

## Supplemental material

These documents can be accessed on the Supplemental material file.
Figure S1: Epidemic curve of cases related to childcare outbreak wave 1 & 2A based on symptom onset date
Figure S2: Epidemic curve of cases related to childcare outbreak wave 1 & 2B based on symptom onset date
Figure S3: Epidemic curve of cases related to childcare outbreak wave 3A based on symptom onset date
Figure S4: Epidemic curve of cases related to childcare outbreak wave 3B based on symptom onset date
Figure S5: Epidemic curve of cases related to childcare outbreak wave 4A based on symptom onset date
Figure S6: Epidemic curve of cases related to childcare outbreak wave 4B based on symptom onset date
Figure S7: Epidemic curve of cases related to childcare outbreak wave 5A based on symptom onset date
Figure S8: Epidemic curve of cases related to childcare outbreak wave 5B based on symptom onset date

## Appendix

Overview of public health measures relevant to childcare in Alberta

While the general audience may not be interested in some of the details outlined in the Appendix, they provide important context to understand the outbreaks. For instance, these details provide context for interpreting the proportion of the population with no vaccines, or help the reader understand who was eligible for polymerase chain reaction (PCR) testing at different times. Documenting policy changes will assist future outbreak epidemiologists in understanding why we found the transmission patterns we did.

The first measure to protect children was implemented in Alberta on March 16, 2020, when childcare facilities including daycares, out-of-school care, and preschools in the province were ordered to close. A limited number of childcare facilities were allowed to re-open on March 23, 2020, to care for children of healthcare and critical infrastructure workers, and this was expanded to include all essential workers on April 1, 2020. Childcare facilities were permitted to open on May 14, 2020, provided facilities could follow guidelines including cohorts of 10 individuals, including staff and children. In Alberta, the staff-to-child ratios in childcare facilities range from 1:3 for infants, to 1:15 for children six years and older, and day homes are limited to six children (not including the day home provider’s children), with not more than three children under three years of age ((21)). The cohort limit was expanded to 30 on June 12, 2020, and removed entirely on August 21, 2020. Guidelines issued also included enhanced screening, hygiene, facility cleaning, staff distancing when possible, and masking when a child became ill. Vaccine requirements were never imposed on childcare staff or eligible children, however, childcare staff were able to book vaccine appointments on May 4, 2021, two days earlier than the general public over age 30, and six days earlier than the general public aged 12 and older. Children aged five to 11 were not eligible for vaccinations until November 26, 2021, and children under five became eligible August 2, 2022.

The PCR testing available for children and childcare workers varied throughout the pandemic. On April 9, 2020, the testing eligibility was expanded to include essential workers who were symptomatic, anyone living with someone 65 years and older, and all Calgary Zone residents. This was quickly expanded to include anyone with symptoms on April 13, 2020. On May 29, 2020, two weeks after childcare facilities were starting to open, testing was expanded to anyone without restrictions. Testing shifted again September 17, 2020, and was focused on only symptomatic cases, known contacts of cases, those linked to outbreaks, and asymptomatic cases in priority locations (which did not include childcare). On December 23, 2021, symptomatic Albertans were asked to use rapid tests and were no longer eligible for PCR testing (with exceptions for those eligible for specific treatments and those in specific high-risk settings). Childcare guidelines were soon shifted to include reference to rapid test results.

Comprehensive screening for COVID-19 variants of concern (VOC) began in early February 2021, coinciding with the arrival of the B.1.1.7 Alpha, and B.1.351 Beta variants in the province ((31)). However, this comprehensive screening was paused when cases were at their peak in waves 3 to 5, and instead VOC screening was prioritized to outbreak-associated cases, hospitalized cases, and a random sample of community cases. Overall, from February 2021 when large scale screening began to the end of our reported period (December 31, 2021), 67% of cases were screened for VOCs (internal data).

Initially, outbreak investigations in childcare settings in Alberta were opened when there was one case confirmed to have COVID-19 ((32)). This shifted to two confirmed cases within 14 days, or two epidemiologically linked cases, in August 2020 ((33)). This definition was updated to only include cases who were at the childcare setting while communicable or who likely acquired there ((34)), and again in September 2021to include outbreaks based on two cases meeting respiratory illness case definition ((35)). Childcare COVID-19 outbreaks stopped being opened on December 31, 2021, and the February 2022 update to the Public Health Management Guidelines did not mention childcare settings beyond that cases should not attend ((36)).

For much of the pandemic, facilities were put on alert and tracking began with a single confirmed case, and publicly reported when there were five or more cases. This changed on March 17, 2021, when public reporting of cases moved to 10 cases. Cases were only included as part of the outbreak if they have a primary connection to the facility (for example, if they work there or attend). For most of the COVID-19 pandemic, when a case was confirmed at a childcare facility in Alberta, the case was referred to a specialist outbreak investigation team to conduct contact tracing.
